# Multiple Roles of SIRT2 in Regulating Physiological and Pathological Signal Transduction

**DOI:** 10.1155/2022/9282484

**Published:** 2022-08-29

**Authors:** Changhui Zhu, Xue Dong, Xiwei Wang, Yingying Zheng, Juanjuan Qiu, Yanling Peng, Jiajun Xu, Zhengbin Chai, Chunyan Liu

**Affiliations:** ^1^Department of Biochemistry and Molecular Biology, School of Basic Medical Sciences, Weifang Medical University, Weifang 261053, Shandong, China; ^2^Medical Research Center, The First Affiliated Hospital of Shandong First Medical University & Shandong Provincial Qianfoshan Hospital, Jinan 250014, China; ^3^Department of Education, The First Affiliated Hospital of Shandong First Medical University & Shandong Provincial Qianfoshan Hospital, Jinan 250014, China; ^4^Department of Anesthesiology, The First Affiliated Hospital of Shandong First Medical University & Shandong Provincial Qianfoshan Hospital, Jinan 250014, China; ^5^Shandong First Medical University & Shandong Academy of Medical Sciences, Jinan 250014, China; ^6^Department of Clinical Laboratory Medicine, Shandong Public Health Clinical Center, Shandong University, Jinan 250102, China; ^7^Innovative Institute of Chinese Medicine and Pharmacy, Shandong University of Traditional Chinese Medicine, Jinan 250355, China

## Abstract

Sirtuin 2 (SIRT2), as a member of the sirtuin family, has representative features of evolutionarily highly conserved nicotinamide adenine dinucleotide (NAD+)-dependent deacetylase activity. In addition, SIRT2, as the only sirtuin protein colocalized with tubulin in the cytoplasm, has its own functions and characteristics. In recent years, studies have increasingly shown that SIRT2 can participate in the regulation of gene expression and regulate signal transduction in the metabolic pathway mainly through its post-translational modification of target genes; thus, SIRT2 has become a key centre in the metabolic pathway and participates in the pathological process of metabolic disorder-related diseases. In this paper, it is discussed that SIRT2 can regulate all aspects of gene expression, including epigenetic modification, replication, transcription and translation, and post-translational modification, which enables SIRT2 to participate in energy metabolism in life activities, and it is clarified that SIRT2 is involved in metabolic process-specific signal transduction mechanisms. Therefore, SIRT2 can be involved in metabolic disorder-related inflammation and oxidative stress, thereby triggering the occurrence of metabolic disorder-related diseases, such as neurodegenerative diseases, tumours, diabetes, and cardiovascular diseases. Currently, although the role of SIRT2 in some diseases is still controversial, given the multiple roles of SIRT2 in regulating physiological and pathological signal transduction, SIRT2 has become a key target for disease treatment. It is believed that with increasing research, the clinical application of SIRT2 will be promoted.

## 1. Introduction

The silent information regulator (sirtuin, SIRT) family, also known as type III histone deacetylases (HDACs), are nicotinamide adenine dinucleotide (NAD+)-dependent deacetylases that are highly conserved from bacteria to humans. To date, seven types of sirtuins have been identified in mammals (SIRT1-7), all of which have different functions and localizations in cells [1]. SIRT1, SIRT6, and SIRT7 mainly exist in the nucleus, where SIRT1 regulates transcription, chromatin modification, and energy metabolism, while SIRT6 and SIRT7 are related to DNA repair and rDNA transcription [2–4]. SIRT3–5 are localized in mitochondria, where they regulate oxidative stress and ATP production in response to caloric restriction [5]. The SIRT2 gene is located on autosomal chromosome 19q13.2, colocalizes with tubulin in the cytosol, and is distributed in metabolically active tissues, such as the liver, heart, skeletal muscle, and brain [6].

Studies have shown that SIRT2 can participate in the regulation of gene expression and regulate signal transduction in metabolic pathways through its post-translational modification of target genes [7, 8]. This paper mainly discusses the specific mechanism by which SIRT2 regulates gene expression in all aspects and through the post-translational modification of each target gene of synthetic metabolism and catabolism in lipid metabolism and glucose metabolism (mainly deacetylation) and directly or indirectly affects the transduction of metabolic-related signals (synergy, etc.), resulting in crosstalk between metabolic signalling pathways; thus, SIRT2 has become a key centre in metabolism. Therefore, SIRT2 can be involved in metabolic disorder-related inflammation and oxidative stress and participate in the pathological process of metabolic disorder-related diseases, rendering SIRT2 a target for the clinical treatment of diseases. This paper first elucidates the specific mechanism of SIRT2 in the correlation between gene expression regulation and metabolic pathway signal transduction to provide new ideas and methods for the treatment of diseases.

## 2. Roles of SIRT2 in Regulating Gene Expression

Gene expression refers to the synthesis of biologically active proteins from the genetic information carried by DNA sequences through replication, transcription, and translation. SIRT2 can regulate gene expression by participating in replication, transcription, translation, epigenetic modification, and post-translational modification.

### 2.1. SIRT2 Regulates Replication, Transcription, and Translation

At the replication level, on the one hand, after DNA damage, SIRT2 mainly recovers DNA replication from the stress state through ataxia telangiectasia-mutated and Rad3-related (ATR) kinase checkpoint. At sites of DNA damage, SIRT2 can deacetylate ATR-interacting protein (ATRIP) K32, thereby driving ATR activation through the binding of ATR-ATRIP to replication protein A-coated single-stranded DNA (RPA-ssDNA). Furthermore, SIRT2 can deacetylate CDK9 lysine 48 and partially depend on ATR to activate CDK9 kinase activity, promoting the recovery of DNA replication [9, 10]. On the other hand, SIRT2 maintains the activity of RNR by deacetylating the subunit RRM2 of ribonucleotide reductase (RNR), ensuring a sufficient supply of precursor dNTPs for DNA synthesis and promoting the progress of DNA replication [11].

At the transcriptional level, SIRT2 inhibits transcription through its deacetylation activity. In the transcription system of mouse cellular RNA polymerase I (Pol I), SIRT2 can deacetylate the TATA box-binding protein (TBP) factor TIF-IB/SL1 TAF_I68_ and impair the binding of TAFI68 to rDNA promoters, resulting in rDNA transcription inhibition [12]. In addition, the interaction between SIRT 2 and the homeobox transcription factor HOXA10 was identified in human cells [13].

At the translational level, the most studied SIRT2 is eukaryotic translation initiation factor 5A (eIF5A). SIRT2 can regulate the subcellular localization of eIF5A by regulating the acetylation level of eIF5A. The acetylated form of eIF5A is located in the nucleus, while unacetylated eIF5A is located in the cytoplasm, which enables it to perform different functions in different cell sites, and its specific functional mechanism remains to be studied [14–16].

### 2.2. SIRT2 Is Involved in Epigenetic and Post-Translational Modifications

After DNA replication, transcription, and translation, the synthesized protein is an inactive protein precursor that needs to undergo various post-translational modifications to become a protein with specific biological functions. Studies have shown that SIRT2 can participate in the epigenetic regulation of genes by the post-translational modification of histones, thereby indirectly regulating life activities within cells. SIRT2 can also directly participate in regulating intracellular life activities through the post-translational modification of nonhistone proteins. The details are shown in Table 1.

In summary, SIRT2 can lead to biologically active proteins through replication, transcription, translation, and epigenetic and post-translational modifications related to gene expression. This finding is consistent with Li et al.'s research, which showed that SIRT2 can target genes and proteins involved in the regulation of gene expression and metabolism and can target certain diseases [31, 32]. This finding suggests that SIRT2 can form crosstalk between gene expression regulation and metabolism and participate in the pathological process of disease.

## 3. Role of SIRT2 in Signal Transduction

### 3.1. SIRT2 and Lipid Metabolism-Related Signal Transduction

**Figure 1:**
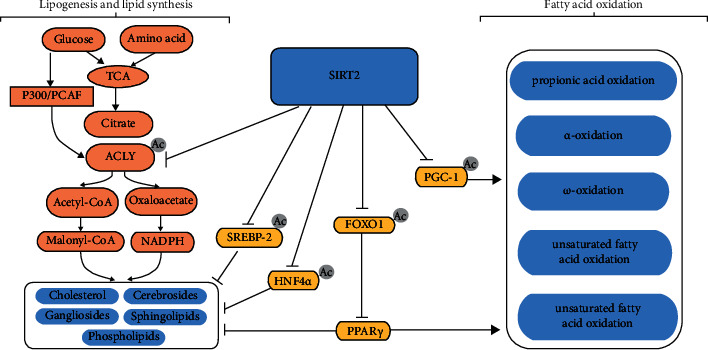
SIRT2 is involved in lipid metabolism. SIRT2 affects lipid synthesis through ACLY. P300/PCAF can stabilize ACLY by acetylation, thereby promoting lipid synthesis. SIRT2 degrades ACLY by deacetylation to inhibit lipid synthesis. SIRT2 promotes the binding of SREBP-2 to the SRE by promoting the nuclear translocation of SREBP-2, thereby promoting the transcription of cholesterol synthesis-related genes and ultimately promoting the synthesis of cholesterol. SIRT2 inhibits lipid synthesis by deacetylating HNF4*α*. SIRT2 promotes fatty acid oxidation and inhibits lipid synthesis by inhibiting the nuclear translocation of FOXO1. In addition, SIRT2 promotes fatty acid oxidation through the deacetylation of PGC-1.

Lipid metabolism includes lipid uptake, synthesis, oxidation, and storage. The key regulatory factors include insulin, glucagon, and adrenaline. Insulin promotes the synthesis of triglycerides in the liver and the storage of triglycerides in white adipose tissue (WAT). Glucagon and adrenaline stimulate the fat decomposition in WAT and fatty acid oxidation in other tissues when nutrients are limited (Figure 1).

#### 3.1.1. Lipogenesis

Studies have shown that inhibiting the expression of SIRT2 can promote fat production. This process involves increasing the expression of many genes associated with adipocyte differentiation, including peroxisome proliferator-activated receptor gamma (PPAR*γ*), CCAAT/enhancer binding protein (C/EBP*α*), glucose transporter 4 (Glut4), adipokine fatty acid-binding protein (aP2), fatty acid synthase (FASN), and C/EBP*β* [33, 34]. For example, in a study by Lu et al., it was shown that targeting and downregulating SIRT2 can lead to the upregulation of FASN and SREBP-1 expression, thereby promoting adipogenesis [35].

PPAR is a key transcription factor that maintains lipid homeostasis in tissues with three isoforms, PPAR*α*, PPAR*β*/*δ*, and PPAR*γ*, which have different subcellular localizations and functions [36]. Among them, PPAR*γ* is a key transcriptional regulator of adipogenesis that can increase the expression of adipogenesis-related genes, including FASN [37]. Studies have shown that mammalian class O (FoxO) forkhead transcription factor 1 (FOXO1) can inhibit the PPAR*γ* gene, leading to the upregulation of SLC2A4 (GLUT4) gene expression, and FOXO1 can also affect GLUT4 to regulate insulin sensitivity [38]. SIRT2 deacetylates FOXO1 and simultaneously reduces the phosphorylation level of FOXO1 by synergistic action (insulin and/or insulin-like growth factor 1 (IGF-1) activates protein kinase B (Akt, PKB) via phosphorylation), preventing FOXO1 from translocating from the nucleus to the cytoplasm, strengthening the binding of FOXO1 to the PPAR*γ* gene promoter, inhibiting transcription of the PPAR*γ* gene, and ultimately inhibiting adipogenesis [39].

#### 3.1.2. Lipid Synthesis

In the de novo synthesis of lipids, ATP-citrate lyase (ACLY) cleaves citrate into acetyl-CoA (acetyl-CoA) and oxaloacetate (OAA) under ATP-consuming conditions, thereby converting intracellular glucose catabolism linked to de novo lipid synthesis of lipids. Therefore, ACLY is a key enzyme in the synthesis of nascent lipids and can regulate gene expression by supplying acetyl-CoA, thereby linking the metabolic status to histone acetylation [40]. Numerous studies have shown that lipid synthesis can be affected by regulating the acetylation state of ACLY through SIRT2 and P300/calcium-binding protein (CBP)-associated factor (PCAF) acetyltransferases. SIRT2 deacetylates ACLY for ubiquitination and degradation, inhibiting de novo lipid synthesis. P300/PCAF catalyses the opposite effect [41, 42]. In addition, studies have shown that SIRT2 can target lysine 458 of hepatocyte nuclear factor 4*α* (HNF4*α*) to deacetylate HNF4*α* to promote its stability, thereby preventing the degeneration of liver fat [43].

Cholesterol synthesis is mainly controlled by the sterol regulatory element binding protein SREBP-2 [44]. In the endoplasmic reticulum, the inactive precursor of SREBP tightly binds the SREBP cleavage activator protein (SCAP) to retain it on the endoplasmic reticulum membrane. In the presence of low sterol levels, the SREBP-SCAP complex is transported to the Golgi apparatus, where the inactive precursor undergoes cleavage by S1P and S2P, converting it into the active form nSREBP-2 (the SREBP-2 nuclear form). Finally, nSREBP-2 translocates to the nucleus and binds the sterol response element (SRE) to activate the transcription of cholesterol synthesis-related genes and promote cholesterol synthesis [45–48]. The binding of nSREBP-2 to the SRE can be mediated by a feedforward mechanism that induces the transcription of SREBP-2 [49]. Studies have shown that SIRT2 can lead to cholesterol biosynthesis by promoting the nuclear translocation of SREBP-2 [50].

### 3.2. Fatty Acid Oxidation

Fatty acid oxidation is the main source of energy in the body and mainly occurs in the liver and muscle but does not occur in nerve tissue. *β*-oxidation and specific oxidation modes (propionic acid oxidation, *α*-oxidation, *ω*-oxidation, and unsaturated fatty acid oxidation) occur.

Recent studies have shown that genes involved in fatty acid oxidation are transcriptionally regulated by PPAR and its coactivator peroxisome proliferator-activated receptor *γ* coactivator 1 (PGC-1). PPAR*α* can enhance fatty acid *β*-oxidation and the expression of ketogenic genes, and PPAR*δ* can induce long-chain fatty acid (LCFA) oxidation genes and control mitochondrial biogenesis and function by inducing PGC-1, thereby affecting the oxidation of fatty acids [51].

Previous studies have shown that SIRT2 is involved in lipid synthesis by affecting the nuclear translocation of FOXO1, which inhibits the transcription of PPAR*γ* [39]. SIRT2 can also inhibit lipid synthesis by downregulating FOXO1 [52]. However, a recent study by Akbulut et al. showed that decreased FOXO1 levels were related to lipid peroxidation and increased SIRT2 expression levels [53]. However, the specific mechanism remains unclear. In addition, Krishnan et al. showed that SIRT2 could deacetylate PGC-1, resulting in increased *β*-oxidation and mitochondrial gene expression [54].

### 3.3. SIRT2 and Glucose Metabolism-Related Signal Transduction

**Figure 2:**
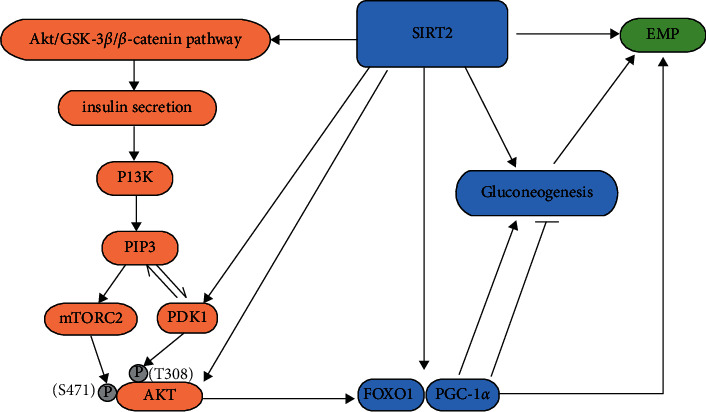
SIRT2 is involved in glucose metabolism. SIRT2 promotes gluconeogenesis by deacetylating FOXO1 and PEPCK. SIRT2 can promote glycolysis by interacting with glycolytic enzymes. SIRT2 can promote insulin secretion through the Akt/GSK-3*β*/*β*-catenin pathway. SIRT2 can also regulate glucose metabolism through the insulin-PI3K-AKT-metabolism pathway. AMPK can phosphorylate and activate SIRT2 and enhance the interaction between SIRT2 and AKT. In addition, SIRT2 can activate AKT and PDK1 through deacetylation, thereby promoting glycolysis and inhibiting gluconeogenesis.

The metabolism of sugar in the human body is divided into anabolism and catabolism, which are in a dynamic balance to stabilize the blood sugar levels. The main processes of glucose metabolism in the human body include the uptake of glucose by intestinal epithelial cells, the decomposition of glycogen in the liver, gluconeogenesis to produce glucose, and the uptake and decomposition of blood glucose by other tissues (Figure 2).

#### 3.3.1. Gluconeogenesis

After fasting, the body is in a state of nutrient deficiency and needs to use substances within the body to produce glucose and provide the energy required for physiological activities. During short-term fasting, the liver produces and releases glucose primarily through glycogenolysis. During prolonged fasting, glycogen is depleted, and hepatocytes use nonsugar substances (lactic acid, pyruvate, glycerol, and glycogenic amino acids) to produce and release glucose through gluconeogenesis.

Studies have shown that SIRT2 is involved in the regulation of gluconeogenesis mainly through phosphoenolpyruvate carboxykinase (PEPCK/PCK), which is encoded by the PCK gene. Since PEPCK can catalyse the conversion of OAA to phosphoenolpyruvate (PEP) in the first step of gluconeogenesis, PEPCK is a key regulatory enzyme involved in hepatic glucose production. Jiang et al. showed that SIRT2 is the main deacetylase that acts on PEPCK1, which reduces the acetylation of PEPCK1 and the association between PEPCK1 and UBR5 (the HECT domain of E3 ubiquitin ligase), increasing the stability of PEPCK1 and thereby promoting gluconeogenesis [55–58]. In addition, SIRT2 can deacetylate FOXO1 and PGC-1*α* and promote the transcription of gluconeogenic enzyme genes, suggesting that SIRT2 may regulate gluconeogenesis through different mechanisms [39, 59].

#### 3.3.2. Glycolysis and Glucose Uptake

After food is ingested, the blood sugar level increases due to the uptake of glucose by epithelial cells in the small intestine. After blood sugar rises, a large amount of energy is produced by consuming glucose to maintain normal physiological activities. After the energy requirement is met, unconsumed glucose is converted into glycogen and converted into fat by stimulating the secretion of insulin.

Hamaidi et al. showed that eight enzymes associated with glycolysis (hexokinase 1 (HK1), phosphofructokinase (PFKP), aldolase A (ALDOA), glyceraldehyde-3-phosphate dehydrogenase (GAPDH), phosphoglycerate kinase 1 (PGK1), enolase 1 (ENO1), pyruvate kinase M (PKM), and lactate dehydrogenase (LDH)) interacted with SIRT2 [60]. Cha et al. further demonstrated that the acetylation levels and activities of glycolytic enzymes (ALDOA, PGK1, ENO1, and GAPDH) were regulated by SIRT2 but not SIRT1 [61]. Therefore, SIRT2 is a key enzyme that regulates the glycolytic pathway. In addition, glucokinase (GCK) can catalyse the conversion of glucose to 6-phosphate glucosamine to facilitate the consumption of glucose via glycolysis. SIRT2 can activate GCK by deacetylating GCK-regulated protein (GKRP), which separates GKRP from GCK (GCK), thereby promoting hepatic glucose uptake [62].

Studies have shown that the inhibition of SIRT2 can not only improve the stability of GKRP protein and promote the degradation of ALDOA but also inhibit glucose-stimulated insulin secretion (GSIS) by regulating the Akt/glycogen synthase kinase (GSK)-3*β*/*β*-catenin pathway in *β* cells, resulting in a decrease in glycolytic flux [63, 64]. The main downstream signalling pathway of insulin action is the phosphatidylinositol 3-kinase (PI3K)-AKT/PKB pathway. Activated AKT can translocate to the nucleus to phosphorylate its targets (FOXO1, etc.), thereby inhibiting gluconeogenesis. In the insulin-PI3K-AKT-metabolism pathway, the activation of AKT first binds to the inositol 1,4,5-triphosphate (PIP (3)) on the plasma membrane, which causing changes in its conformation to activate kinase PDK1 and rapamycin complex 2 (mTORC2), and then, PDK1 and mTORC2 phosphorylate AKT Thr 308 and Ser 471 to activate AKT, so that the activated AKT is translocated to the nucleus [33, 39, 65–67]. SIRT2 can deacetylate AKT and PDK1, thereby promoting their membrane localization and activation [68, 69]. Insulin induces the dissociation of SIRT2 from AKT because adenosine 5′-monophosphate-activated protein kinase (AMPK) activity is negatively regulated by insulin. The insulin-induced activation of PI3K-AKT inhibits AMPK, reduces the phosphorylation of SIRT2 by AMPK, and reduces SIRT2 activity to induce a dissociation between SIRT2 and AKT. Glucose and nutrient degradation and PI3K inhibition promote SIRT2 binding to AKT [58, 70, 71]. Therefore, while the decrease in SIRT2 reduces the secretion of insulin and the activation of AKT, the interaction between SIRT2 and AKT can be increased by the negative regulation of AMPK by insulin to inhibit gluconeogenesis.

#### 3.3.3. Role of SIRT2 in the Intersection between Lipid and Glucose Metabolism

As previously mentioned, ACLY links glucose catabolism with lipid synthesis, creating crossover between lipid and glucose metabolism. SIRT2 can deacetylate ACLY to promote its ubiquitin-mediated degradation and reduce the conversion of glucose into acetyl-CoA, thereby inhibiting lipid synthesis [41, 42, 72]. Furthermore, while ACLY expression is regulated by SREBP-1, its activity is regulated by the PI3K/Akt pathway [73]. Studies have shown that ACLY is also a target of Akt, which can directly phosphorylate and activate ACLY [74]. SIRT2 can interact with Akt and regulate AKT activation through the insulin-PI3K-AKT pathway [68]. Therefore, SIRT2 can also indirectly act on ACLY through the PI3K-AKT pathway, thereby affecting the metabolism of carbohydrates and lipids to maintain metabolic stability in the body.

## 4. SIRT2 and Metabolic Disorders

### 4.1. Inflammation

Research has shown that there is a close relationship between metabolism and immunity [75]. Systemic low-grade chronic inflammation plays a key role in the pathogenesis of metabolic disorders, and the main regulators are nuclear factor-*κ*B (NF-*κ*B), mitogen-activated protein kinase (MAPK), and the inflammasome (NOD-like receptor pyrin domain-containing 3, NLRP3).

In the NF-*κ*B pathway, SIRT2 is inactivated by Lys 310 deacetylation of the NF-*κ*B subunit p65 in the cytoplasm, which decreases the expression of its downstream target genes (interleukin-1*β* (IL-1*β*), IL-6, monocyte chemoattractant protein 1 (MCP-1), a chemokine that regulates the expression and secretion of activated normal T cells (RANTES), matrix metalloproteinase 9 (MMP-9), and MMP-13), thereby exerting anti-inflammatory effects [76, 77]. Lee et al. showed that inhibiting SIRT2 could reduce the phosphorylation and degradation of NF-*κ*B, reduce the phosphorylation of NF-*κ*B subunit p65 Ser 536, reduce the nuclear translocation of p65, and inhibit the expression of NF-*κ*B [78]. This cascade reduces the expression of iNOS and NO, the downstream target genes of NF-*κ*B, and exerts an anti-inflammatory effect. However, there has been no discussion regarding the specific molecular mechanism linking the phosphorylation and acetylation of p65. In other experimental inflammatory disease models, the anti-inflammatory effect of SIRT2 has been demonstrated by inhibiting the NF-*κ*B signalling pathway [78–81]. However, Pais et al. showed that SIRT2 also stimulates the activation of NF-*κ*B through Toll-like receptors (TLRs), leading to an inflammatory response [82]. In addition, studies have shown that the inflammatory NF-*κ*B pathway is associated with SIRT2 and HSP90, but current research related to this topic is still controversial [83, 84].

In the MAPK-related inflammatory signalling pathway, SIRT2 deacetylates MKP-1, decreasing the acetylation level of MKP-1, which promotes the transduction of TLR signalling, upregulates MAPK signalling, and increases the ERK, p38, and JNK phosphorylation levels, thereby promoting the production of the proinflammatory factors IL-1*β*, IL-6, TNF-*α,* and iNOS, leading to the occurrence of inflammation [85–88].

In the NLRP3 inflammatory signalling pathway, NLRP3 is acetylated by macrophages, which promotes the assembly and activation of the NLRP3 inflammasome, thereby enabling the production of the inflammatory cytokines IL-1*β* and IL-18 to induce inflammatory reactions [89]. By contrast, the targeted deacetylation of NLRP3 by SIRT2 inactivates this process.

### 4.2. Oxidative Stress

SIRT2 protects against oxidative damage, and SIRT2-mediated deacetylation of its targets plays an important role during oxidative stress [90, 91]. Studies have shown that reactive oxygen species (ROS) are important enzymes that catalyse the generation of oxygen free radicals and activate oxidative stress responses [92]. SIRT2 can act on ROS through signalling pathways, such as FOXO3, Nrf2, PEPCK1, poly (ADP-ribose) polymerase 1 (PARP1), and p65, to reduce the generation of oxygen free radicals [93].

Under oxidative stress, SIRT2 can activate its target FOXO3 through deacetylation, and then, FOXO3a shuttles to the nucleus and induces the expression of its target antioxidants manganese superoxide dismutase (MnSOD), SOD1/2, p27 (Kip1), and Bim, which reduce the cellular ROS levels and enhance the cellular antioxidant capacity [94–96].

Nrf2 is a key transcription factor that regulates the gene expression of multiple antioxidant enzymes [97]. Under normal physiological conditions, Nrf2 stably binds Kelch-like enoyl-CoA hydratase-associated protein 1 (Keap1) and is stabilized in the cytoplasm, preventing Nrf2 translocation to the nucleus and inhibiting the ability of Nrf2 to activate target genes [98]. Under oxidative stress, SIRT2 induces the dissociation of Nrf2 and Keap1, and Nrf2 translocates to the nucleus to interact with antioxidant response elements (AREs), thereby activating the target gene antioxidant element haem oxygenase 1 (HO-1) to protect cells from oxidative damage [97–100].

In addition, SIRT2 can regulate ROS by targeting metabolic enzymes, such as glucose 6-phosphate dehydrogenase (G6PD), phosphoglyceride mutant enzyme (PGAM2), and NF-*κ*B [101]. Under oxidative stress conditions, SIRT2 can deacetylate and activate the key enzyme G6PD in the pentose phosphate pathway (PPP) to stimulate the production of cytoplasmic NADPH, thereby maintaining the reduced form of GSH against oxidative damage [102–104]. In addition, Xu et al. showed that SIRT2 could increase NADPH production through the deacetylation and activation of PGAM2 to enable cells to respond to oxidative stress [105]. SIRT2 promotes the activation of enzymes, including NADPH oxidase, xanthine oxidoreductase, inducible NO synthase, cyclooxygenase-2, and cytochrome p450, through the activation of NF-*κ*B. SIRT2 can also target ROS-inhibiting enzymes, such as SOD 1 and 2, thioredoxin, and glutathione S-transferase, which increase the level of ROS in cells. In turn, NF-*κ*B activity is also regulated by the ROS levels [82, 106].

Studies have shown that SIRT2 can also deacetylate PARP1 K249, resulting in the ubiquitination and degradation of PARP1 by WW domain-containing protein 2 (WWP2), thereby alleviating the vascular oxidative stress injury caused by PARP1 mediated by angiotensin II (Ang II) [107]. Alternatively, SIRT2 can deacetylate K431 of Bcl-2-associated athanogene 3 (BAG3), thereby mediating BAG3 binding to PARP1 to promote the ubiquitination degradation of WWP2 at PARP1 K249 to protect against oxidative damage [108].

Collectively, these findings suggest that SIRT2 plays a key role in regulating the oxidative stress response, which protects organisms from metabolic damage through oxidative stress-dependent mechanisms.

## 5. Role of SIRT2 in Disease

**Figure 3:**
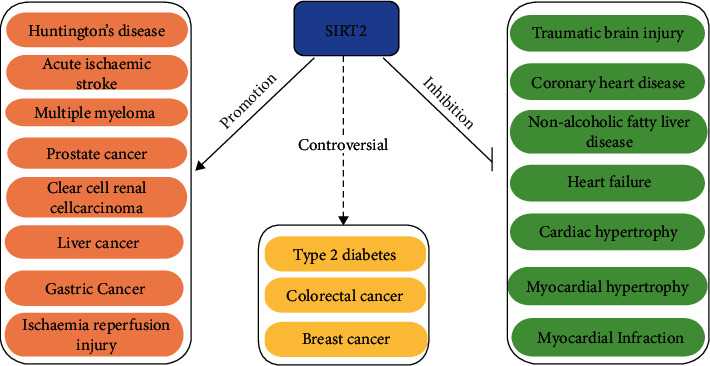
The role of SIRT2 in various metabolic disorders. SIRT2 promotes the progression of Huntington's disease, acute ischaemic stroke, multiple myeloma, prostate cancer, clear-cell renal cell carcinoma, liver cancer, gastric cancer, and ischaemia reperfusion injury. SIRT2 inhibits the progression of traumatic brain injury coronary heart disease, nonalcoholic fatty liver disease, heart failure, cardiac hypertrophy, myocardial hypertrophy, and myocardial infarction. The role of SIRT2 in type 2 diabetes, colorectal cancer, and breast cancer remains controversial.

SIRT2 participates in metabolic pathways through various signalling pathways and is associated with the occurrence of nervous system-related degenerative diseases, various tumours, diabetes, and cardiovascular diseases (Figure 3).

### 5.1. SIRT2 and Neurodegenerative Diseases

Neurodegenerative diseases are neurasthenic disorders in which the main pathogenic mechanism is protein degradation and inflammatory defects caused by impairments in energy metabolism and disturbances in redox homeostasis [109, 110]. SIRT2 is expressed in almost all brain cells, particularly oligodendrocytes in the central nervous system, and is indirectly involved in cellular processes relevant to the pathophysiology of neurodegenerative diseases [111, 112].

In Huntington's disease (HD), SIRT2 inhibition was shown to prevent HD by reducing sterol biosynthesis and exerting neuroprotective effects [113]. This protective effect was closely related to the transcriptional regulation of genes that control metabolism, including sterol and fatty acid biosynthesis, carbohydrate metabolism, and purine metabolism [114].

Traumatic brain injury (TBI) is associated with an increased risk of different neurodegenerative diseases, and the main pathological features include neuroinflammation, oxidative stress, excitotoxicity, and mitochondrial dysfunction. Studies have shown that SIRT2 plays a protective role in TBI. In TBI, SIRT2 inhibition stimulates NF-*κ*B activation, induces MMP-9 expression, and perturbs the blood-brain barrier (BBB) integrity, allowing plasma components, such as macrophages and inflammatory factors, to more readily cross the BBB, which worsens neuroinflammation [115]. Alternatively, SIRT2 exerts a protective effect by inhibiting p53-induced ferroptosis [116].

The pathogenesis of acute ischaemic stroke (AIS) mainly involves oxidative stress, impaired mitochondrial function, and poststroke inflammation [117]. Studies have shown that SIRT2 expression is positively correlated with the severity of AIS and proinflammatory cytokines, but its specific mechanism still needs further study.

### 5.2. SIRT2 and Cancer

Metabolic pathway reprogramming is a hallmark of cancer, and SIRT2 can affect tumour progression through metabolic pathways. In recent years, the role of SIRT2 in cancer has remained controversial, and SIRT2 acts as both an oncogene and a tumour suppressor; to a certain extent, its impact may be greater than that of SIRT1 [118]. Therefore, SIRT2 has become a new focus of tumour research.

As a tumour-promoting factor, SIRT2 mainly promotes the immune escape of tumour cells by inducing immune avoidance, improving energy metabolism, and changing the tumour microenvironment, thereby inducing the development of tumours. In this article, we mainly describe the effects of SIRT2 on cancer through metabolic pathways. Studies have shown that SIRT2 can promote various cancers through various metabolic pathways. In gastric cancer, SIRT2, as a downstream target of miR-138, can increase PEPCK1-related metabolism, thereby promoting gastric cancer cell migration and invasion through the RAS/ERK/JNK/MMP-9 pathway [119, 120]. In colorectal cancer (CRC), Wnt/*β*-catenin inhibition can increase the expression level of the Wnt/*β*-catenin target gene SIRT2 and promote the proliferation of CRC cells [121]. In breast cancer, SIRT2 can be activated by NOTCH signalling, leading to the deacetylation and activation of high aldehyde dehydrogenase (ALDH1A1), thereby promoting breast cancer progression [122]. In liver cancer, SIRT2 can promote mitochondrial metabolism and inhibit the E-cadherin pathway, thereby promoting cancer cell invasion [123]. In prostate cancer (PCa), SIRT2-mediated deacetylation of LIFR-K620 inhibits the progression of PCa [124]. In multiple myeloma (MM), a high expression of SIRT2 can act on the RAS/ERK signalling pathway to promote MM cell proliferation, thereby promoting MM progression [125]. In clear-cell renal cell carcinoma (ccRCC), SIRT2 deacetylates G6PD and increases its stability, thereby promoting ccRCC progression [126]. In addition, the deacetylation of lactate dehydrogenase (LDH) by SIRT2 increases its enzymatic activity, leading to the accumulation of lactate and promoting tumour cell proliferation [127]. Under conditions of nutrient excess, the activity of SIRT2 decreases, and the level of the acetylation of PKM2 increases, which increases its enzymatic activity and decreases lactate production and pyruvate accumulation, thereby resulting in a similar Warburg effect to promote cancer progression [128–130].

As a tumour suppressor, SIRT2 can inhibit cancer by not only inhibiting fibroblast activity and angiogenesis but also inhibiting cancer through various metabolic pathways [131]. Studies have shown that T cells from SIRT2-deficient mice exhibit increased glycolysis and oxidative phosphorylation, resulting in enhanced proliferation and effector functions and exerting antitumour effects [60]. In breast cancer, SIRT2 not only promotes tumours but also suppresses tumours. For example, SIRT2 can inhibit the antioxidant activity of peroxiredoxin-1 (Prdx-1), rendering breast cancer cells sensitive to ROS-induced DNA damage and cytotoxicity, leading to apoptosis in breast cancer cells [132]. Alternatively, SIRT2 regulates the reversible acetylation of PHGDH through TIP60 and promotes the binding of PHGDH and RNF5 to induce PHGDH degradation, which reduces the serine and glycine levels and disturbs redox homeostasis, thereby inhibiting breast cancer cell proliferation [133]. In colorectal cancer, SIRT2 also inhibits CRC progression [134]. SIRT2 deacetylation is an important mechanism that regulates IDH1, which can exhibit tumour suppressor function by targeting the IDH1 K224 residue [135]. Furthermore, in many other tumour cell types, SIRT2 can deacetylate ACLY and reduce its stability, thereby inhibiting tumour cell proliferation [41].

In brief, the current evidence suggests that SIRT2 plays dual roles in cancer, and its specific role in cancer still needs to be revealed by further studies.

### 5.3. SIRT2 and Diabetes

In recent years, with the increasing number of patients with type 2 diabetes (T2D), increasing attention has been given to its pathogenesis and treatment. Studies have shown that T2D pathogenesis is mediated by abnormal glucose metabolism, insulin resistance (IR), and *β*-cell function. SIRT2 is a key hub in metabolic processes and, therefore, has been investigated as a potential therapeutic target for T2D [62, 90].

PEPCK1 can promote the utilization of glucose and glutamine via anabolism; thus, it is an important marker in evaluating T2D [119]. Studies have shown that inhibiting the deacetylase SIRT2 in the liver can promote the acetylation of PEPCK1, leading to its degradation and thereby inhibiting gluconeogenesis [56, 57].

IT in skeletal muscle is associated with a prediabetic state [136]. SIRT2 expression decreases with the progression of diabetic osteoarthritis (OA), resulting in increased H3 acetylation, oxidative stress, and inflammatory responses. Inhibiting SIRT2 accelerates the progression of diabetic OA, whereas the upregulation of SIRT2 may alleviate the development of diabetic OA by inhibiting the oxidative stress and inflammatory responses associated with H3 deacetylation. Furthermore, SIRT2 attenuates oxidative stress and mitochondrial dysfunction and increases insulin sensitivity in hepatocytes [137].

Impaired hepatic glucose uptake (HGU) contributes to postprandial hyperglycaemia in T2D. In HFD-fed obese diabetic mice and db/db mice, SIRT2 activity decreased with decreasing hepatic NAD + levels, which enhanced GKRP acetylation and impaired HGU, whereas K126 deacetylation of GKRP alleviated glucose tolerance and impaired IR. Therefore, SIRT2 ameliorated HGU damage by deacetylating K126 of GKRP, dissociating glucose-dependent GCK from GKRP [62, 63].

These studies show that SIRT2 protects against diabetes. However, Zheng et al. showed the opposite effects, suggesting that the haplotype of SIRT2 contributes to transcription factor binding and T2D susceptibility [138]. Although the role of SIRT2 in diabetes is currently controversial, undoubtedly, SIRT2 may be a novel molecular target for future diabetes treatment.

### 5.4. SIRT2 and Cardiovascular Disease

Currently, cardiovascular disease is an important factor in human mortality, and its pathogenesis involves oxidative stress, the inflammatory response, apoptosis, autophagy, myocardial ischaemia-reperfusion, ageing, and energy restriction [139]. Therefore, studies have increasingly been conducted to explore the relationship between SIRT2 and cardiovascular disease.

Atherosclerosis is a main cause of cardiovascular disease. Studies have shown that during atherosclerosis, SIRT2 can inhibit the occurrence and development of atherosclerotic macrophages by inhibiting the polarization of macrophages [139]. Alternatively, SIRT2 can directly act on its target Nrf2/FOXO3/PARP1 and indirectly act on PARP1 by deacetylating BAG3, which alleviates the further aggravation of hypertension and oxidative stress, prevents intraluminal occlusion and stenosis from causing organic lesions, and avoids the occurrence of coronary heart disease (CHD) of myocardial ischaemia [97, 98, 107, 108]. Furthermore, in acute myocardial infarction (AMI), functional DNA sequence variants (DSVs) can alter the SIRT2 levels by affecting the transcriptional activity of the SIRT2 promoter, leading to the progression of AMI into severe disease [140].

In heart failure, the downregulation of the creatine kinase (CK) system is a hallmark of impaired myocardial energy in failing hearts, and the acetylation of muscle creatine kinase negatively affects high-energy phosphate transfer in heart failure [141]. Deacetylation by SIRT2 reduces the acetylation of key lysine residues, improves dimer formation, and restores CK muscle form (CKM) activity in failing heart tissue, resulting in the amelioration of heart failure. In addition, miR-140-5p inhibitor experiments have shown that inhibiting miR-221-3p leads to SIRT2 activation, thereby targeting Nrf2/FOXO3, inhibiting cardiomyocyte apoptosis and oxidative stress, and relieving cardiac exhaustion [142, 143].

In cardiac hypertrophy, SIRT2 acts as a cardioprotective deacetylase [144]. SIRT2 binds and deacetylates LKB1 at lysine 48, promoting the phosphorylation of LKB1 and its translocation from the nucleus to the cytoplasm, which activates LKB1-AMPK signalling. Some other targets of SIRT2, such as FOXO1 and PGC1-*α*, are also known regulators of cardiac hypertrophy. FOXO1, FOXO3a, and PGC1*α* are also downstream of LKB1-AMPK signalling. Therefore, the activation of LKB1-AMPK signalling may be a core mechanism underlying SIRT2-mediated cardioprotective effects. This hypothesis was confirmed in a study by Mei et al., who showed that CSN6 was partially dependent on the stabilization of the Nkx2.2 protein, which is a transcriptional repressor of SIRT2 that inhibits SIRT2 and promotes cardiac hypertrophy [145]. Gu et al. showed that PHD finger protein 19 (PHF19) can inhibit the expression of SIRT2, thereby promoting Ang II-induced cardiomyocyte hypertrophy [146]. Furthermore, in myocardial infarction, maintaining SIRT2 activity can interfere with the interaction between CDK5 and SIRT2 through the interaction between LncHrt and SIRT2, thereby activating the downstream LKB1-AMPK cascade to maintain the balance of cardiac metabolism [147]. Collectively, these studies have shown that SIRT2 is a cardioprotective deacetylase in cardiovascular disease and could be a therapeutic target for cardiovascular disease.

### 5.5. SIRT2 and Other Metabolic Diseases

Recent studies have shown that the NAD+/SIRT2 pathway plays an important role in regulating nonalcoholic fatty liver disease (NAFLD) [148]. NR (nicotinamide riboside) stimulates SIRT2, promotes SIRT2 deacetylation, and stabilizes Fndc5 to prevent HFD-induced NAFLD; SIRT2 also deacetylates and stabilizes CCAAT/enhancer-binding protein beta (C/EBP*β*) to prevent NAFLD; alternatively, NAFLD can be treated by targeting the SIRT2-HNF4*α* pathway [43, 149, 150]. Furthermore, during hepatic ischaemia-reperfusion (I/R) injury, SIRT2 upregulates the MAPK pathway through MKP-1 deacetylation, thereby enhancing the inflammatory response and cell death, leading to the exacerbation of postoperative liver injury [151].

### 5.6. Application of SIRT2 in Disease

As previously mentioned, SIRT2 enables crosstalk between gene expression regulation and metabolism and plays an important role in related diseases; thus, it can be used as a key target for disease treatment or drug development. In mouse models of HD and acute cerebral ischaemia, AK-7, an inhibitor of SIRT2, showed neuroprotective effects [152, 153]. The cardiotoxicity caused by the antineoplastic drug doxorubicin can be alleviated by increasing SIRT2 activity with limonoid or the adiponectin agonist ADP355 [142, 154]. In the treatment of NAFLD, silibinin can increase the activity of SIRT2, thereby protecting the liver [155].

In addition, numerous studies have shown that SIRT2 has different expression characteristics in different diseases; thus, it can be used as a biomarker of disease to judge the occurrence, development, and prognosis of disease. In tumours, SIRT2 can be used as a biomarker for the clinical diagnosis and treatment of tumours, such as to predict the development of the epithelial-mesenchymal transition (EMT), hepatocellular carcinoma (HCC), and endometrial cancer (EC) [156–158]. SIRT2 predicts future atrial fibrillation prevalence in coronary artery disease (CAD) and myocardial infarction [159, 160].

Overall, currently, SIRT2 is mainly applied to screen drugs as therapeutic targets for disease or as a biomarker to facilitate the diagnosis and prognostic evaluation of disease. However, the clinical application of SIRT2 requires further research.

## 6. Conclusion

The current study shows that SIRT2 can form crosstalk between gene expression regulation and metabolic regulation, and thus, SIRT2 plays an important role in maintaining metabolic homeostasis and the occurrence of related diseases. SIRT2 can participate in metabolic processes, such as adipogenesis, lipid synthesis, gluconeogenesis, glycolysis and glucose uptake, inflammatory response, and oxidative stress, through the regulation of gene expression and cause neurodegeneration, cancer, diabetes, cardiovascular disease, metabolic disorders, etc. It is believed that in-depth research in the future could help us elucidate more molecular mechanisms of SIRT2 between gene expression and metabolic pathways to use it as a potential target for disease treatment and promote its clinical application.

## Figures and Tables

**Table 1:** Posttranslational modifications involving SIRT2.

Types of post-translational modifications	Types of catalytic proteins	SIRT2 catalytic effect	Function
Acetylation (Kac)	Histone non histone	Deacetylation	Regulates transcription and diverse biological processes [17–20]
Methacrylation (Kmea)	Histone	Removes H3K18mea	Enables crosstalk between metabolism and epigenetic regulation, and the specific mechanism needs further study [21]
Crotonylation ((Kcr))	Histone	Removes Kcr	Regulates transcription [22, 23]
Benzoylation (Kbz)	Histone	Removes histone Kbz	Regulates transcription [24, 25]
Gamma-oxononanoylation (Kgon)	Histone	Removes histone Kgon	Interferes with histone assembly into nucleosomes [26, 27]
4-Oxononanoylation (4-ONylation)	Histone	Removes 4-ONyl	Prevents nucleosome assembly under oxidative stress [28]
Lipoylation	Nonhistone	Delipoylation	Regulates cell metabolism [29]
Myristoylation	Nonhistone	Demyristoylates ADP-ribosylation factor 6 (ARF6) K3	Promotes ARF6 activation [30]

## Data Availability

All the data generated or analysed during this study are included in this published article.
